# Obesity, Underweight, and Smoking Are Associated with Worse Cardiorespiratory Fitness in Finnish Healthy Young Men: A Population-Based Study

**DOI:** 10.3389/fpubh.2017.00206

**Published:** 2017-08-18

**Authors:** Georgios Nikolakaros, Tero Vahlberg, Kari Auranen, Lauri Sillanmäki, Takis Venetoklis, Andre Sourander

**Affiliations:** ^1^General Psychiatry Outpatient Clinic, Psychiatric Care Division, Satakunta Hospital District, Pori, Finland; ^2^Department of Mathematics and Statistics, University of Turku, Turku, Finland; ^3^Research Center for Child Psychiatry, University of Turku, Turku, Finland; ^4^Department of Social Policy, University of Turku, Turku, Finland

**Keywords:** cardiorespiratory fitness, exercise test, non-linear models, obesity, overweight, physical fitness, smoking, thinness

## Abstract

**Background:**

Obesity and smoking are strongly associated with worse cardiorespiratory fitness (CRF). Most previous studies that have examined the association of body composition with CRF have neither assessed non-linearity nor separately examined the effects of underweight. Thus, very little is known on how underweight affects CRF. Possible joint effects of obesity and smoking on CRF have not been adequately explored.

**Aims:**

We examined the association between body mass index (BMI) and smoking with CRF in 1,629 Finnish army conscripts. We focused on non-linear effects of BMI in order to assess the importance of underweight. We also examined whether the cooccurrence of obesity and smoking potentiates their deleterious effects on CRF.

**Methods:**

We used the Cooper’s 12-minute run test (12MR) to measure CRF. The 12MR score was analyzed as continuous (linear, polynomial, and restricted cubic spline regression) and categorical. In categorical analyses, we used binary logistic regression with the 12MR score in two groups (low = lowest quintile vs. intermediate/high = quintiles 2–5) and multinomial logistic regression with the 12MR score in three groups (low = lowest quintile, intermediate = quintiles 2 and 3, and high = quintiles 4 and 5).

**Results:**

Non-linearity in the spline model was statistically significant (*p* < 0.001). In addition, the non-linear models had a clearly better fit than the linear one in terms of Akaike Information Criterion and *R*-squared values. There was a statistically significant interaction between smoking and BMI (*p* < 0.01). In the categorical analysis, overweight/obese regular smokers were at a particularly high risk of not achieving high CRF.

**Conclusion:**

In healthy young men, not only overweight/obesity but also underweight may be associated with worse CRF. This provides a potential mechanism for the previously reported association between underweight and increased mortality. The cooccurrence of overweight/obesity and regular smoking may have a deleterious effect on CRF.

## Introduction

The body mass index (BMI), defined as weight (kg) divided by height squared (m^2^), is widely used to classify body composition according to the World Health Organization criteria as normal weight (18.5–24.9), underweight (<18.5), overweight (25.0–29.9), and obesity (≥30.0). Obesity, overweight, and underweight are associated with increased mortality (1).

Cardiorespiratory fitness (CRF) can be assessed by measuring exercise performance under standardized conditions, either in the laboratory (2) or with a field test like the Cooper’s 12-minute run test (12MR) (3–6). The 12MR measures the distance the subject is able to run in 20 minutes (6). The 12MR has a good overall reliability in estimating CRF in young healthy men, but it might underestimate CRF at low values and overestimate CRF at high values (5). The 12MR has been used to assess CRF in American and Austrian military personnel (6, 7), in Finnish male conscripts (3), and in Brazilian male firefighters (4). In addition, the 12MR has been used to show a decrease in the average CRF of Finnish conscripts during the last decades (8). A low CRF is an independent risk factor for increased mortality (9, 10) and morbidity (9, 11). CRF is decreased in obesity (12, 13), overweight (13), and in smokers (3, 12). Some studies have suggested that in selected study populations CRF is decreased in the underweight too (14–17). Most previous studies on the association between BMI and CRF have used BMI as a continuous variable or have not used a separate category for the underweight. These approaches assume a linear association between BMI and CRF, or no association between underweight and CRF. To the best of our knowledge, there are no previous studies on the association between both BMI and smoking with CRF in healthy young adults with a wide range of CRF.

We used both linear and non-linear methods to examine the association between BMI and smoking with CRF among 1,629 Finnish male conscripts. We particularly assessed the association between underweight and CRF and examined possible synergistic effects of obesity and smoking on CRF.

## Materials and Methods

### Study Population

**Figure 1:**
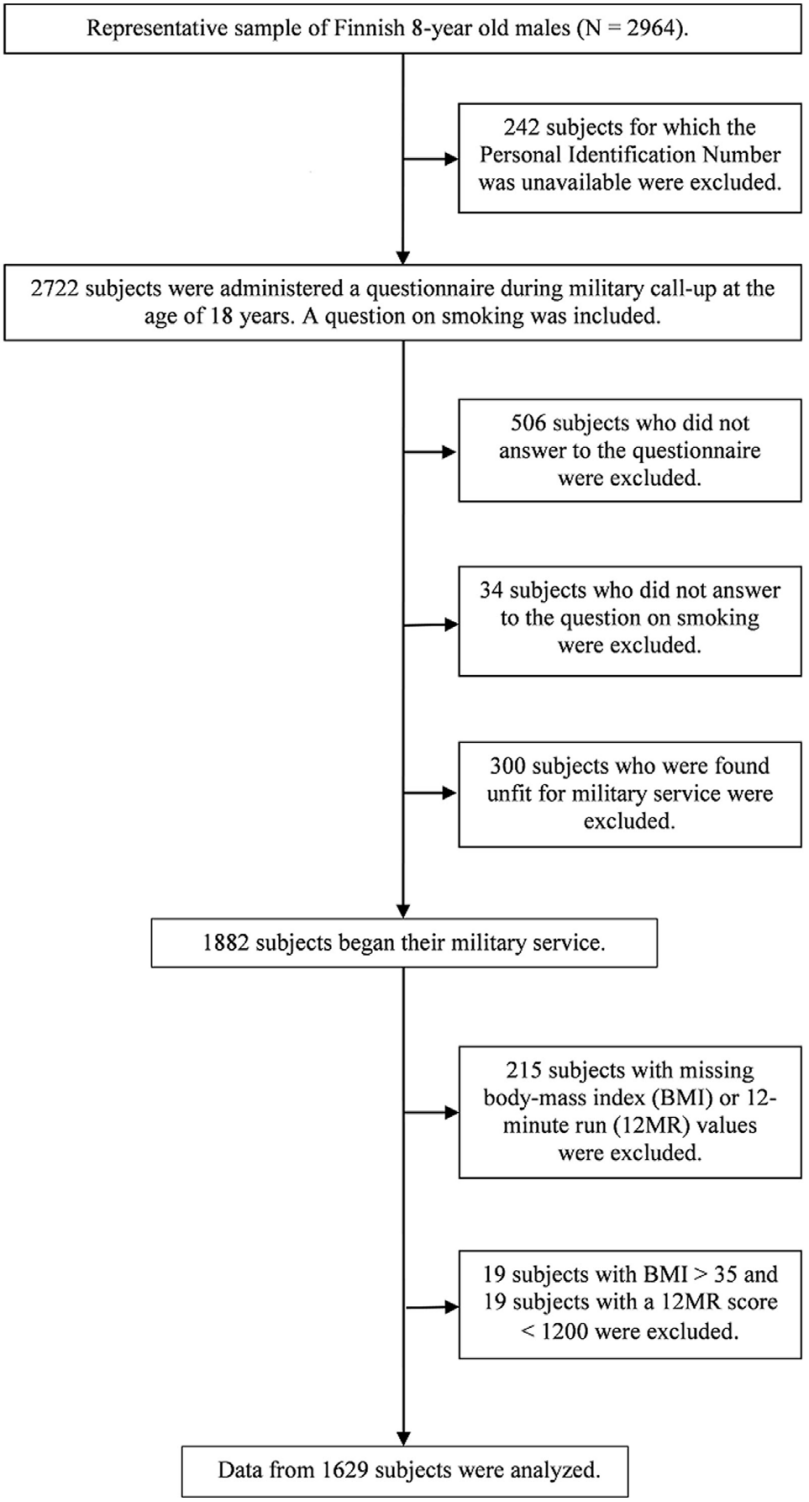
Flow chart showing the selection of the study subjects.

The study population came from our “From Boy to a Man” project (18). The initial cohort comprised 2,964 males, a representative sample of Finnish males born in 1981 that were first examined at the age of 8 years. At the military call-up when around 18 years old, 2,216 subjects were re-examined with a questionnaire that contained a question about smoking in five categories: no smoking, occasional smoking, smoking 1–5 cigarettes per day, 6–10 cigarettes per day, and ≥11 cigarettes per day (19). Of these subjects, 1,882 began their military service. In Finland, all conscripts undergo a 12MR during the first 3 weeks of service and their height and weight are measured. Details of the measurements have been previously reported (3). We obtained the BMI and the 12MR values from the Finnish military. The final study cohort consisted of 1,629 subjects. Figure 1 shows the selection of study subjects. We excluded subjects with a 12MR score of less than 1,200 m to reduce the effect of lack of motivation. We also excluded subjects with a BMI value of 35 or more, because obesity of such a high degree is often sufficient to exempt from military service.

### Statistical Analysis

Age was examined as a continuous predictor. BMI was examined as a continuous predictor, and as a categorical predictor in three categories (underweight, normal weight, and overweight/obese) with cutoff points at 18.5 and 25. To enhance statistical power, the “smoking 1–5 cigarettes per day,” “smoking 6–10 cigarettes per day,” and “smoking ≥11 cigarettes per day” groups were merged to a “regular smoker” group, and smoking was examined with one-way ANOVA in three categories: non-smoker, occasional smoker, and regular smoker.

The 12MR score was first studied as a continuous outcome variable. We compared three models: (a) a linear regression model with BMI as the continuous predictor, (b) a polynomial regression model with a second-order BMI term, and (c) a restricted cubic spline regression model with three knots at BMI values of 18.5, 25, and 30. We compared the models using their *R*-squared values, and the Akaike Information Criterion (AIC) (20). The restricted cubic spline regression analysis was built as follows (expected 12MR score *y_i_* for individual *i*):

(1) $$E\text{(}y_{i}\text{)}=\;\beta_{\text{0}}\;+\;\beta_{\text{1}}x_{i}\;+\;\beta_{\text{2}}z_{\text{1}}{\;}_{i}\;+\;\beta_{\text{3}}z_{\text{2}}{\;}_{i}\;+\;\beta_{\text{4}}v\text{(}x_{i}\text{),}$$

where *x_i_* is the BMI level for individual *i, z*_1_*_i_*, and *z*_2_*_i_* are indicator variables for the individual being in smoking categories 2 or 3, and *v*(*x_i_*) is the value of the restricted cubic spline at BMI value *x_i_*. The spline function (including the linear term) was constructed as explained in Desquilbet and Mariotti (21). The model was then extended by adding the interaction between BMI and smoking:

(2) E(yi)=β0+β1xi+β2z1i+β3z2 i+β4v(xi)+ψ1xiz1i +ψ2v(xi)z1i+ψ3xiz2i+ψ4v(xi)z2i.

Non-linearity in the effect of BMI on the 12MR score was examined with an *F* test for β_4_ = 0 vs. β_4_ ≠ 0 in model (1). The presence of interaction between BMI and smoking was tested by comparing models (2) and (1) with an *F* test, the null hypothesis being ψ_1_ = ψ_2_ = ψ_3_ = ψ_4_ = 0 (i.e., no interaction).

The interaction between BMI and smoking was statistically significant. Thus, we proceeded to further examine the 12MR score as a categorical outcome variable. We analyzed the association between BMI and 12MR score separately among non-smokers, occasional smokers, and regular smokers, and the association between smoking and 12MR score separately among underweight, normal weight, and overweight/obese subjects. In these separate analyses, we used both binary and multinomial logistic regression for the categorized 12MR score: subjects in the lowest 12 MR score quintile were considered having low CRF, those in the second or third quintile intermediate CRF, and those in the fourth or fifth quintile high CRF. The categorical analyses also allowed us to differentiate between two different outcomes: having a low CRF and not achieving a high CRF. We used exact logistic regression in the occasional smoker/underweight group because there were no observations in the lowest quintile of the 12MR score. A two-tailed *p* < 0.05 was considered statistically significant. Statistical analyses were performed using SAS version 9.4 (SAS Institute, Inc., Cary, NC, USA), and R version 3.1.3 (R Foundation for Statistical Computing, Vienna, Austria). Analyses that included age as a covariate gave similar results and are not presented.

### Ethics Approval and Consent to Participate

Participation in the study was voluntary. Written informed consent was obtained from the parents at baseline and from the boys at follow-up. The study was approved by the Joint Commission on ethics of Turku University Hospital and Turku University. The Finnish Defense Forces gave permission to use the data on BMI and 12MR.

## Results

Table 1 shows summary measures and frequency distributions of age, BMI, smoking, and 12MR score.

**Table 1:** Summary measures and frequency distributions of age, BMI, smoking, and 12MR score.

Characteristic	Mean (SD)	Median	Minimum	Maximum	*N* (% of subjects)
Age	19.52 (0.81)	19.43	17.48	24.79	
BMI (continuous)	22.47 (3.27)	21.91	15.35	34.88	
BMI (categorical)					
Underweight			15.35	18.50	130 (8.0)
Normal weight			18.51	24.96	1,191 (73.1)
Overweight/obese			25.00	34.88	308 (18.9)
Smoking					
No smoking					689 (42.3)
Occasional smoking					395 (24.3)
Regular smoking					545 (33.5)
12MR score (continuous)	2,533.1 (336.4)	2,550.0	1,200	3,740	
12MR score (categorical)					
Low (first quantile)			1,200	2,260	325 (20.0)
Intermediate (quantiles 2 and 3)			2,265	2,620	653 (40.1)
High (quantiles 4 and 5)			2,625	3,740	651 (40.0)

*12MR, Cooper’s 12-minute run test; BMI, body mass index; N, number of subjects*.

### Analyses with the 12MR Score As a Continuous Outcome Variable

#### Age

In linear regression analysis, age had a significant negative association with the 12MR score (*p* = 0.007). The variation in 12MR score explained by age was very small, the *R*-squared value was 0.004.

#### Body Mass Index

**Figure 2:**
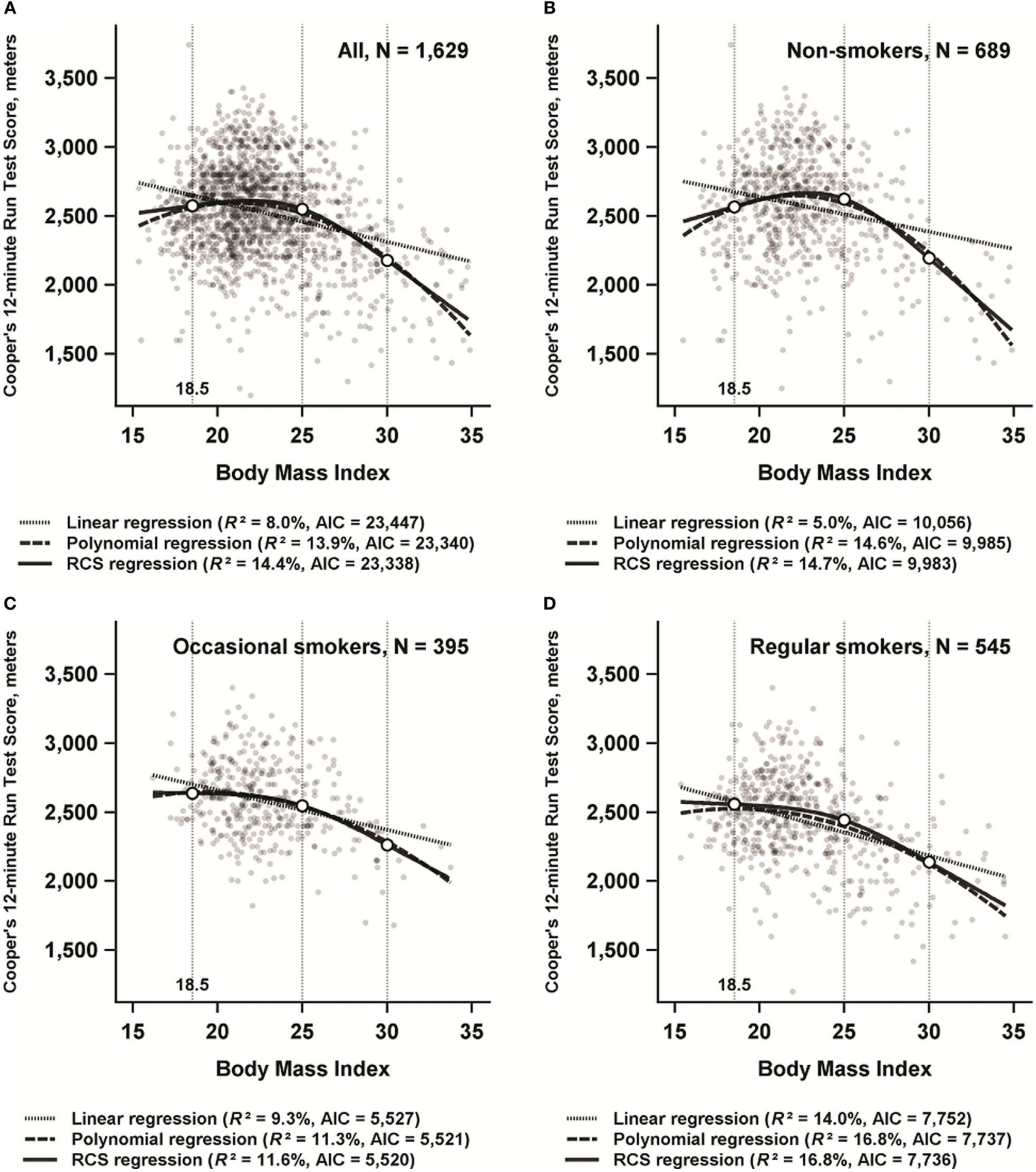
Linear regression, polynomial regression with second-order term, and restricted cubic splines (RCS) regression analyses with Cooper’s 12-minute run score as outcome variable and body mass index (BMI) as predictor variable among 1,629 participants in the “From a boy to a man” study, born in 1981 in Finland. In the RCS regression model, knots were placed at BMI values of 18.5, 25, and 30. Akaike Information Criterion (AIC) and *R*-squared (*R*^2^) values were calculated separately for each model. **A** = All subjects, **B** = Non-smokers, **C** = Occasional smokers, **D** = Regular smokers.

Figure 2A shows the plots and the AIC and *R*-squared values of the three regression models in the whole study population. The curves of the non-linear models were similar to each other, and visual comparison with the linear model supported non-linearity. The AIC and *R*-squared values of the linear model showed a clearly worse fit compared to the non-linear models. In the restricted cubic spline regression model, non-linearity was statistically significant (*p* < 0.001), and the model containing the interaction term between BMI and smoking was superior to the model containing only the main effects (*p* < 0.01).

#### Smoking

The mean 12MR score in non-smokers, occasional smokers, and regular smokers was 2,579.2 m [95% confidence interval (CI) 2,554.6–2,603.8], 2,583.2 m (95% CI 2,550.7–2,615.7), and 2,438.6 m (95% CI 2,411.0–2,466.3), respectively. In one-way ANOVA, the effect of smoking was significant, *F*(2, 1,626) = 34, *p* < 0.001. In *post hoc* analyses with Bonferroni correction, the difference between regular smokers and both non-smokers and occasional smokers was statistically significant (*p* < 0.001), whereas the difference between non-smokers and occasional smokers was not (*p* > 0.99).

#### BMI in Different Smoking Categories

Figures 2B–D show the plots of the three regression models separately for non-smokers, occasional smokers, and regular smokers. Non-linearity was most evident in non-smokers, but the association between BMI and the 12MR score was the strongest among regular smokers. Non-linearity was statistically significant in all three groups (*p* < 0.001 for non-smokers and regular smokers, and *p* < 0.01 for occasional smokers).

### Analyses with the 12MR Score As a Categorical Outcome Variable

Occasional smokers were less likely to have low or high CRF and more likely to have intermediate CRF compared to non-smokers (*p* < 0.001, χ^2^ test).

#### Logistic Regression with BMI As a Predictor Variable Separately in Each Smoking Category

Table 2 shows the results of the binary logistic regression (low CRF vs. intermediate/high CRF). Underweight was associated with low CRF only among non-smokers. Overweight/obesity was associated with low CRF in all smoking categories. Table 3 shows the results of the multinomial logistic regression (high vs. low CRF and high vs. intermediate CRF). Underweight was associated with lower odds of achieving high CRF when compared to low CRF in non-smokers only, and when compared to intermediate CRF in non-smokers and regular smokers. Overweight/obesity was associated with lower odds of achieving high CRF when compared to both low CRF and intermediate CRF in all smoking categories, but contrary to the binary model the association was clearly the strongest among regular smokers.

**Table 2:** Binary logistic regression analysis, with the 12MR score as outcome variable and BMI as the predictor variable.

	*N*	OR	95% CI
**Non-smokers**			
BMI			
Normal weight^a^ (18.5 ≤ BMI < 25)	510	1.00	
Underweight (BMI < 18.5)	61	2.86	1.46–5.43
Overweight/obese (BMI ≥ 25)	118	4.85	3.02–7.79
**Occasional smokers (exact test)**			
BMI			
Normal weight^a^ (18.5 ≤ BMI < 25)	300	1.00	
Underweight (BMI < 18.5)	18	0.42	0.00–2.02
Overweight/obese (BMI ≥ 25)	77	3.44	1.68–6.96
**Regular smokers**			
BMI			
Normal weight^a^ (18.5 ≤ BMI < 25)	381	1.00	
Underweight (BMI < 18.5)	51	0.89	0.36–1.96
Overweight/obese (BMI ≥ 25)	77	5.59	3.49–9.04

*The ORs denote odds of belonging to the lowest quintile (low CRF) compared to quintiles two to five (intermediate/high CRF). Separate analyses by smoking*.

*12MR, Cooper’s 12-minute run test; BMI, body mass index; CI, confidence interval; CRF, cardiorespiratory fitness; N, number of subjects; OR, odds ratio*.

*^a^Reference category*.

**Table 3:** Multinomial logistic regression analysis, with the 12MR score as outcome variable and BMI as the predictor variable.

	Low CRF			Intermediate CRF		
	*N*	OR	95% CI	*N*	OR	95% CI
**Non-smokers**						
BMI						
Normal weight^a^	65	1.00		166	1.00	
Underweight^b^	18	4.07	2.02–8.2	24	2.12	1.13–3.99
Overweight/obese^c^	49	6.37	3.80–10.7	36	1.83	1.10–3.05
**Occasional smokers^d^**						
BMI						
Normal weight^a^	26	1.00		131	1.00	
Underweight	0	0.40	0.00–2.04	8	0.87	0.29–2.5
Overweight/obese	19	6.07	2.62–14.3	41	2.63	1.38–5.2
**Regular smokers**						
BMI						
Normal weight^a^	74	1.00		172	1.00	
Underweight	9	2.05	0.76–5.5	34	3.34	1.50–7.4
Overweight/obese	65	16.9	7.39–38.8	41	5.00	2.00–10.6

*The ORs denote odds of belonging to the lowest quintile (low CRF) or to the second or third quintile (intermediate CRF), compared to belonging to the fourth of fifth quintile (high CRF). Separate analyses by smoking*.

*12MR, Cooper’s 12-minute run test; BMI, body mass index; CI, confidence interval; CRF, cardiorespiratory fitness; N, number of subjects; OR, odds ratio*.

*^a^Normal weight (18.5 ≤ BMI < 25) is the reference category*.

*^b^BMI < 18.5*.

*^c^BMI ≥ 25*.

*^d^Exact test*.

#### Logistic Regression with Smoking As a Predictor Variable Separately in Each BMI Category

Table 4 shows the results of the binary logistic regression (low CRF vs. intermediate/high CRF). Regular smoking was associated with higher odds of low CRF among normal weight and overweight/obese subjects. Occasional smoking was associated with lower odds of low CRF in underweight and overweight/obese subjects. Table 5 shows the results of the multinomial logistic regression (high vs. low CRF and high vs. intermediate CRF). Regular smoking was associated with lower odds of high CRF compared to both low and intermediate CRF particularly among overweight/obese subjects. Occasional smoking was associated with higher odds of achieving high CRF when compared to intermediate CRF in normal weight and overweight/obese subjects, and with lower odds of achieving high CRF when compared to low CRF in underweight subjects.

**Table 4:** Binary logistic regression analysis, with the 12MR score as outcome variable and smoking as the predictor variable.

	*N*	OR	95% CI
**Underweight (BMI < 18.5)^a^**			
Smoking			
No smoking (reference)	61	1.00	
Occasional smoking	18	0.01	0.00–0.49
Regular smoking	51	0.52	0.18–1.37
**Normal weight (18.5 ≤ BMI < 25)**			
Smoking			
No smoking (reference)	510	1.00	
Occasional smoking	300	0.65	0.40–1.05
Regular smoking	381	1.65	1.15–2.37
**Overweight/obese (BMI ≥ 25)**			
Smoking			
No smoking (reference)	118	1.00	
Occasional smoking	77	0.46	0.25–0.87
Regular smoking	113	1.91	1.31–3.22

*The ORs denote odds of belonging to the lowest quintile (low CRF) compared to quintiles two to five (intermediate/high CRF). Separate analyses by BMI*.

*12MR, Cooper’s 12-minute run test; CI, confidence interval; BMI, body mass index; N, number of subjects; OR, odds ratio*.

*^a^Exact test*.

**Table 5:** Multinomial logistic regression analysis, with the 12MR score as outcome variable and smoking as the predictor variable.

	Low CRF			Intermediate CRF		
	*N*	OR	95% CI	*N*	OR	95% CI
**Underweight (BMI < 18.5)^a^**						
Smoking						
No smoking (reference)	18	1.00		24	1.00	
Occasional smoking	0	0.08	0.00–0.44	8	0.64	0.18–2.2
Regular smoking	9	1.18	0.32–4.41	34	3.32	1.16–10.3
**Normal weight (18.5 ≤ BMI < 25)**						
Smoking						
No smoking (reference)	65	1.00		166	1.00	
Occasional smoking	26	0.78	0.48–1.28	131	1.54	1.14–2.09
Regular smoking	74	2.35	1.59–3.48	172	2.14	1.59–2.88
**Overweight/obese (BMI ≥ 25)**						
Smoking						
No smoking (reference)	49	1.00		36	1.00	
Occasional smoking	19	0.75	0.34–1.66	41	2.21	1.06–4.6
Regular smoking	65	6.25	2.56–15.3	41	5.37	2.12–13.6

*The ORs denote odds of belonging to the lowest quintile (low CRF) or to the second or third quintile (intermediate CRF), compared to belonging to the fourth of fifth quintile (high CRF). Separate analyses by BMI*.

*12MR, Cooper’s 12-minute run test; BMI, body mass index; CI, confidence interval; CRF, cardiorespiratory fitness; N, number of subjects; OR, odds ratio*.

*^a^Exact test*.

## Discussion

In this population-based sample of young men assessed as healthy for military service, underweight and overweight/obesity were associated with lower CRF. This non-linear association was modified by smoking, with overweight/obese regular smokers having a particularly low odds ratio of achieving high CRF.

A non-linear association between BMI and CRF has been previously suggested by studying BMI as a categorical variable (16), and by using polynomial regression in college students (14) and school youth in middle (17) and late (15) adolescence. However, Sekulić et al. (14) studied non-obese physically active subjects, and in the study by Hung et al. (16) subjects were 18–60 years old. In these previous studies, the effects of smoking were not taken into account.

We used polynomial regression, and also spline regression, which avoids the former’s potential problems (autocorrelation of terms, dominance of the area around the peak). Both non-linear models were clearly superior to the linear model, and non-linearity in the spline model was statistically significant. Our results suggest that when modeling the association of BMI with CRF, BMI should neither be used as a linear continuous predictor nor should underweight be included in the normal weight group in categorical BMI analysis.

In our data, BMI explained around 14% of 12MR variation. By comparison, in the study of non-obese physically active subjects by Sekulić et al. (14) and BMI explained 10% of CRF variation. In the study of Taiwanese youth aged 9–18 years (15), non-linearity was present only among 16–18 years old boys, and BMI explained 7.4% of CRF variation. Taken together, these results suggest that as children enter adolescence and early adulthood non-linearity between body composition and CRF gradually emerges and becomes more pronounced. Such a pattern has been suggested by Lu et al. (22), but the 50-m run test used in this study may not measure specifically CRF. Alternatively, the non-linearity of the association between BMI and CRF among adolescents has been attributed to lack of systematic sports training (17). In a study from the Cooper Center, BMI and physical activity were strong determinants of CRF, but independent associations with other factors were much weaker (2). This strong effect of BMI further stresses the importance of distinguishing underweight as its own category to avoid bias in the analyses of other factors.

Our underweight subjects had worse CRF than those with normal weight. Since physical activity is a major determinant of CRF (2), low physical activity could explain the association between underweight and low CRF. This is supported by the association of underweight with low physical activity in previous studies (23, 24). Studies with data on BMI, physical activity, and CRF are needed to explore fully such potential associations.

Underweight is associated with increased mortality (1, 25), also among subjects underweight already at the age of 20 years (26). The mechanisms underlying this association have not been fully elucidated (27, 28), but a recent study has found an excess of deaths due to external causes (25). In countries where food and medical care are frequently unavailable, undernutrition and chronic disease may be involved, but these potential factors are not relevant to our study cohort. Since CRF and mortality have similar associations with underweight, we speculate that low CRF in early adulthood might explain the association between underweight and mortality later in life. Results from studies on physical activity (which is strongly associated with CRF) support this notion. Underweight Puerto Rican men had a reduced excess mortality if they were physically active (29). In a large meta-analysis, adjusting for physical activity attenuated the effect of underweight on mortality (30). It has been shown that the underweight have a higher mortality risk irrespective of smoking (25). We found that underweight was associated with worse CRF most consistently in non-smokers, but partly also in regular smokers. We do not have an explanation for this finding. Future studies should further explore possible associations between smoking, underweight, and low CRF.

In this dataset, the combination of overweight/obesity and regular smoking greatly increased the odds of not achieving high CRF. Cooccurrence of low CRF with other cardiovascular risk factors has been previously shown (31). Results by Stea et al. (12) also suggest that overweight/obesity, regular smoking, and low CRF often occur together in young adulthood. Clustering of cardiovascular risk factors is associated with a high incidence of atherosclerosis in young adults (32), and low CRF has an adverse effect on mortality over and above the effect of overweight and obesity (33). Therefore, CRF should be part of a comprehensive assessment of cardiovascular risk factors already in young adulthood (34).

Previous studies have reported a detrimental effect of smoking on CRF (3, 12). We found that in normal weight and overweight/obese subjects regular smoking was associated with lower CRF. The mean 12MR score of occasional smokers was similar to the one of the non-smokers. This is in accordance with a previous study of Finnish conscripts (3). In this study, occasional smokers were more probable to have a moderate level of physical activity and less probable to be physically active or passive compared to non-smokers. This is compatible with our finding of relatively fewer occasional smokers among subjects with both low and high CRF when compared to intermediate CRF. Thus, occasional smokers may be a heterogeneous group. Occasional smoking has received little attention in medical research. In a previous study of the same cohort, we have shown that psychopathology during childhood has a much stronger association with regular smoking than with occasional smoking in young adulthood (19). It would be interesting to examine respective associations with CRF.

Cardiorespiratory fitness has often been analyzed as a binary variable, with the lowest quintile representing low fitness (10, 35). However, CRF might have a more graded effect on mortality and morbidity (11). We found that the interaction between BMI and CRF was partly confined to high CRF; overweight/obese regular smokers were particularly unlikely to achieve high CRF, whereas they were not particularly likely to have low CRF. Thus, different factors and corresponding health behaviors may be involved in avoiding low CRF vs. achieving high CRF. This has clear implications for future research, as well as for public health goals on the optimal amount of physical activity and avoidance of obesity and smoking.

Our study has several limitations. First, we used BMI to assess body composition. Information on waist circumference and measures of fat-free mass would have allowed a more comprehensive picture of adiposity. However, in a previous study substituting BMI with waist circumference did not substantially increase the amount of variation in CRF explained by the model (2). In addition, in a recent study, BMI was not inferior to waist circumference or skinfold measurements in predicting the 12MR score (4). We used measured height and weight, and thus avoided potential bias from using reported values. Second, we had full information on only 55% of the original cohort. However, around 10% of the subjects were excluded because they were found unfit for military service, which does not decrease validity since we extrapolate results to healthy subjects. We excluded men with a BMI of 35 or more, to reduce selection bias. Thus, our results do not cover men with severe obesity. Third, our sample size was not large enough to examine separately overweight and obesity, and regular smoking of different intensity. Fourth, we obtained information only at one point in time, which precludes etiological inferences. Finally, we did not have information on women, physical activity, or whether current non-smokers had been smoking before. However, in a previous study of Finnish conscripts only 2.8% of the subjects were ex-smokers (3). Our study also has several strengths. Height, weight, and the 12MR score were measured under standardized conditions, and the study subjects were motivated to obtain a good 12MR score (3). The different modeling approaches revealed consistently a non-linearity in the association between BMI and CRF.

This study shows that the association of BMI with CRF among young men is not linear, but both underweight and overweight/obesity are associated with lower CRF. Overweight/obese regular smokers are at particularly high risk of not achieving high CRF. More research is needed to examine the relationship of social and mental health factors with underweight, overweight, obesity, smoking, and CRF.

## Ethics Statement

Participation in the study was voluntary. Informed consent was obtained from the parents at baseline and from the boys at follow-up in accordance with the Declaration of Helsinki. The study was approved by the Joint Commission on ethics of Turku University Hospital and Turku University. The Finnish Defense Forces gave permission to use the data on BMI and 12MR.

## Author Contributions

GN conceived of the study, reviewed the literature, designed and performed statistical analyses, and wrote the first draft of the manuscript. TeroV and KA designed, performed, and supervised statistical analyses. LS designed and performed statistical analyses. TakisV provided statistical advice. AS conceived of the study and provided overall supervision. All authors interpreted results and contributed to the final version of the manuscript. All authors have approved the final manuscript.

## Conflict of Interest Statement

The authors declare that the research was conducted in the absence of any commercial or financial relationships that could be construed as a potential conflict of interest.
